# Longitudinal Impact of Physical Activity on Brain Pulsatility Index and Cognition in Older Adults with Cardiovascular Risk Factors: A NIRS Study

**DOI:** 10.3390/brainsci11060730

**Published:** 2021-05-31

**Authors:** Hanieh Mohammadi, Christine Gagnon, Thomas Vincent, Ali Kassab, Sarah Fraser, Anil Nigam, Frédéric Lesage, Louis Bherer

**Affiliations:** 1 Montreal Heart Institute, Montreal, QC H1T 1C8, Canada; hanieh.mohammadi@polymtl.ca (H.M.); christine.gagnon@icm-mhi.org (C.G.); thomas.tv.vincent@gmail.com (T.V.); anil.nigam@icm-mhi.org (A.N.); frederic.lesage@polymtl.ca (F.L.); 2 Department of Medicine, University of Montreal, Montreal, QC H3T 1J4, Canada; 3 Research Center, University of Montreal Health Centre, Montreal, QC H2X 3E4, Canada; ali.kassab@umontreal.ca; 4 Interdisciplinary School of Health Sciences, Faculty of Health Sciences, University of Ottawa, Ottawa, ON K1N 6N5, Canada; Sarah.Fraser@uottawa.ca; 5 Institute of Biomedical Engineering, Polytechnique Montreal, Montreal, QC H3T 1J4, Canada; 6 Institut Universitaire de Gériatrie de Montréal, Montreal, QC H3W 1W4, Canada

**Keywords:** near-infrared spectroscopy, cerebral pulsatility, longitudinal, physical activity, cardiovascular risk factors

## Abstract

Recent studies have shown that optical indices of cerebral pulsatility, including cerebral pulse amplitude, are linked to cerebrovascular health. A chronically higher cerebral pulsatility is associated with cognitive decline. Although it is widely known that regular physical activity improves cognitive functions, little is known about the association between physical activity and the optical index of cerebral pulsatility. This study assessed the impact of 12 months of regular physical activity on the changes in the optical index of cerebral pulsatility and explored its association with cognition. A total of 19 older adults (aged 59–79 years) with cardiovascular risk factors (CVRF) completed the study. Low-intensity, short-duration walking as a brief cardiovascular challenge was used to study the impact of regular physical activity on post-walking changes in cerebral pulsatility index. The participants walked on a gym track while a near-infrared spectroscopy (NIRS) device recorded hemodynamics data from the frontal and motor cortex subregions. Our data indicated that 12 months of physical activity was associated with lower global cerebral pulse amplitude, which was associated with higher cognitive scores in executive functions. Further, the global cerebral pulsatility index was reduced after short-duration walking, and this reduction was greater after 12 months of regular physical activity compared with the baseline. This may be an indication of improvement in cerebrovascular response to the cardiovascular challenge after regular physical activity. This study suggests that 12 months of physical activity may support cognitive functions through improving cerebral pulsatility in older adults with CVRF.

## 1. Introduction

The heartbeat generates pulsatile blood flow and pressure [1]. As the blood travels towards the brain, the arterial tree cushions these fluctuations in blood flow and pressure [2,3]. This leads to a mostly steady blood flow to the neurons, even during diastole, when the heart is relaxed [1]. With aging, stiffened arteries diminish the ability to cushion these pulsatile oscillations; hence, the distal cerebral microvessels are burdened with higher flow pulsations [2]. Previous research has indicated that the presence of cardiovascular risk factors (CVRF) can exacerbate the transmission of the higher pulsatility to cerebral circulation [4]. A chronically higher pulsatility in the blood flow that supplies the neurons is thought to be a potential risk factor contributing to cerebral microvascular damage and cognitive decline [5].

Research shows that regular physical activity improves cardiovascular functions [6,7]. Even individuals with CVRF with cognitive impairment improved their cognitive performance with regular physical activity [8]. This improvement in cognitive functions could be explained by the benefits of exercise on vascular health [9]. This includes a decrease in the indices of arterial stiffness [10] and an improvement in post-exercise vascular responses [11,12] . One may hypothesize that regular physical activity improves the functioning of the arterial walls, perhaps by improving the arterial response to pulsatility. Hence, more pulsatility is cushioned by the arterial walls, which reduces the pulsatility that reaches the brain and then translates into a reduction in cerebral pulsatility, which could support cognitive functions. Therefore, assessing pulsatility in the brain microvasculature, as well as tracking its changes with regular physical activity and its association with cognitive performance may help to further understand the underlying mechanisms of cognitive decline and its improvement through physical activity.

Physical activity temporarily escalates systolic blood pressure and imposes higher stress on the arterial walls [13]; likely, with physical activity compared to the resting state, the impairment in the damping of pulsatility is more pronounced. Hence, it is possible that a brief cardiovascular challenge allows us to identify changes in pulsatility that are not apparent in the resting state. Therefore, we sought to study the impact of long-term physical activity on changes in cerebral pulsatility immediately after a brief cardiovascular challenge. Among cardiovascular challenges, we chose to work on low intensity and short duration walking, as it is a common daily task for older individuals [11].

There are several non-invasive tools to quantitatively assess cerebral pulsatility. These techniques, such as carotid [14,15] and transcranial Doppler [16,17] ultrasound, phase-contrast magnetic resonance imaging (PC-MRI) [5], pulse wave velocity (PWV) [18], pulse plethysmography [19] and blood pressure [20] are often confined to probing larger vessels. The data acquired with these techniques introduce a single, global index of pulsatility and often do not assess the spatial distribution of pulsatility in the cerebral microcirculation [11]. Also, some of these techniques, such as PC-MRI, are not portable and have experimental limitations in the study of physical activity.

In recent years, near-infrared spectroscopy (NIRS) has been used to study cerebral pulsatility [21,22,23,24,25]. These studies reported that the optical index of cerebral pulsatility, namely cerebral pulse amplitude, is sensitive to cerebrovascular health. Cerebral pulse amplitude quantifies the instantaneous expansion of the arterial walls as a result of the passing pulse pressure wave [22,23] and it is related to the stiffness of the arteries in the regions probed by NIRS sources and detectors [22]. Tan et al. (2017) showed that, with age-related stiffening of the arteries, the cerebral pulse amplitude increases [23]. Further, Tan et al. (2016) showed that cerebral pulsatility indices can track changes in cerebrovascular tone both globally and regionally in voluntary breath-holding and in the Sternberg memory task [25]. Nevertheless, to the best of our knowledge, there is no study that has explored the effects of long-term physical activity on changes in cerebral pulse amplitude, its changes with short-duration walking and its association with cognitive performance.

The objectives of the present study were threefold: (1) Assess the impact of 12 months of physical activity on cerebral pulsatility index extracted from NIRS data (before walking). We hypothesized that cerebral pulse amplitude would decline with 1 year of regular physical activity. (2) Assess whether this reduced cerebral pulsatility index (before walking) is associated with improvement in cognitive performance. It was expected that reduction in the cerebral pulse amplitude would be correlated with improving behavioral cognitive scores. (3) Assess the impact of regular physical activity on changes in the cerebral pulsatility index with a brief cardiovascular challenge (before walking versus after walking). It was expected that the changes in cerebral pulse amplitude would be greater after 1 year of regular physical activity compared to the baseline.

## 2. Materials and Methods

### 2.1. Participants and Screening Procedure

A total of 43 participants with CVRF were originally enrolled in this observational study. From this population, 21 participants provided complete datasets (see exclusion and inclusion criteria). 17 participants had NIRS at T0 (start of the experiment) but not at T12 (end of the experiment at 12 months); 3 participants had missing blood test parameters (see Section 2.2 and Section 2.4); 1 participant had missing Stroop test results (see Section 2.3), and 1 participant had a missing PASE score (see Section 2.4). Hence, data for the remaining 21 participants were analyzed, from which 2 participants were excluded from further analysis due to insufficient NIRS data quality (see Section 2.9). Therefore, the data for the remaining 19 older adults were used for the analyses, and their demographic characteristics are summarized in Table 1. Our study population (*n* = 19), age ranged from 59–79 years, with a mean age of 68.05 ± 4.99 years, and 68.4% were women. Participants were regular members of the preventive medicine and physical activity center (EPIC Centre) of the Montreal Heart Institute in Montreal, Canada. Inclusion criteria were right-handed older adults; native French speakers; ability to walk independently on the gym track; the presence of CVRF such as age, high total cholesterol, diabetes, hypertension, low high-density lipoprotein (HDL), high low-density lipoprotein (LDL), cigarette smoking; and all required blood test parameters to calculate the Framingham score (see Section 2.4), PASE score, NIRS data and Stroop test results for both time points of T0 and T12. The exclusion criteria were incomplete NIRS data; missing blood test parameters, PASE score or Stroop test results; moderate to severe valvular heart disease; congestive heart failure; a recent history of myocardial infarction; previous head injury; major psychiatric illness; use of central-nervous-system-affecting medication; uncontrolled hypertension; and alcoholism (more than 2 drinks per day). Approval of all aspects of this study was obtained from the Montreal Heart Institute’s research ethics (#120304). All participants provided written informed consent prior to their participation and could drop out at any time.

### 2.2. Blood Draw and Blood Pressure Measurement

Blood samples were collected from the participants at time points T0 and T12. The blood draw was performed following overnight fasting, using a protocol similar to [26,27]. The blood test parameters were fasting glucose, total cholesterol, LDL and HDL. The average blood pressure was determined, using a digital sphygmomanometer, after the participant had rested for 5 min in a seated position.

### 2.3. Cognitive Assessment

Participants completed the Stroop test, which was extracted from the Delis-Kaplan executive functions system battery [28]. This test was completed at time points T0 and T12 and included four conditions, (1) color naming of rectangles (printed in red, green or blue); (2) reading of color-meaning words printed in black ink (reading the words blue, red or green); (3) inhibition, i.e., refraining from reading the word and naming the color of the ink the word is printed in, which is incongruent (e.g., the word blue, printed in green ink); and (4) switching between color naming and reading of color-meaning words that are printed in incongruent ink color. For all four conditions, participants were instructed to perform the task as quickly as possible and make as few mistakes as possible. The first two Stroop conditions assess processing speed, whereas the two latter conditions assess executive functions (inhibition and switching). Response times were recorded, as well as corrected and non-corrected errors, which were then summed to determine total errors for each condition of the Stroop test. The results of the Stroop test response times are reported in the Table 2.

### 2.4. Assessment of the CVRF and Intensity of Physical Activity

Framingham scores were used to assess the burden of CVRF on the participants. This score combines scores of sex, age, cholesterol or LDL, HDL, blood pressure, diabetes mellitus and smoking status to estimate the 10-year risk of developing coronary heart disease (CHD) events [29]. Due to the unavailability of Framingham age-scores for individuals older than 74 years, the age-scores for those five participants older than this age were linearly extrapolated. The physical activity level in the participants were quantified using the PASE (physical activity scale for older adults) questionnaire [30]. Scores on the PASE range from 0 to 793, and a higher score indicates a greater level of physical activity. In addition, physical activity intensity was assessed using the Borg scale of perceived exertion [31]. The range of the Borg scale is from 1 to 10, and a higher score indicates greater intensity of physical activity.

### 2.5. NIRS Device and Optodes Configuration

**Figure 1:**
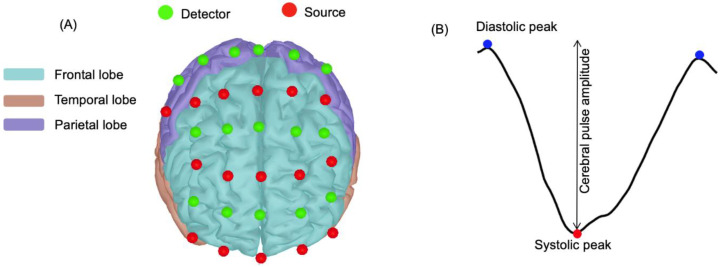
(**A**) Frontal view of the source and detector placements on the Colin27 template. Red dots represent the NIRS sources, and green dots represent detectors. The source and detector arrangement covered frontal and parietal cortex subregions. (**B**) Example of a single cerebral pulse waveform for a channel of a participant. The change in intensity between the systolic (red dot) and the diastolic (blue dots) peaks represents cerebral pulse amplitude (adapted from [24]). *x*-axis is time (s) and *y*-axis is NIRS signal intensity.

The NIRS device was an in-house-developed portable system described in Lareau et al. (2011) [32] and has been previously validated with a commercial NIRS device [33]. It has 16 near-infrared avalanche light-emitting diode (LED) sources and 16 detectors. It generates near-infrared light at 735 and 850 nm and records the data at 20 Hz. The NIRS optodes were built for the NIRS experiment with mobility in mind; hence, the design of the optodes featured a spring that provided stable contact with the scalp and boosted light-coupling efficiency. The optodes were orthogonal to the head surface and were fixed on a socket using a stretchable band, and they were mounted on a long-lasting, flexible mesh to account for the head contours of the participants. The NIRS helmet was placed on the participants’ heads according to the anatomical positions in the international 10–20 EEG system [34]. A triaxial accelerometer was placed on the helmet to help determine mobility periods and motion artefacts of the participants during data analysis. The data from the accelerometer and the detectors were sent to the computer in real time using a Bluetooth device. A LabView user interface was used to calibrate the system, start and stop acquisitions, manage task triggers and visualize the recorded data. The design of the source and detector configuration included the frontal cortex and the subregions of motor cortex, as presented on the cortex of Colin27 template in Figure 1A using Brainstorm [35]. The basic functions of each brain lobe are presented in [36]. The frontal cortex is the area sensitive to age-related cerebral hemodynamic changes, which has been shown to improve as a result of regular physical activity [37,38]. Furthermore, the frontal cortex has been reported to be a good target when exploring vascular changes in the aging brain [39].

### 2.6. Walking Paradigm for NIRS Recording

Participants were instructed to walk on the ground at a self-selected pace [40] in a room where markings indicated a 10 m long path to follow. Participants walked for two distinct trials, identified as blocks 1 and 2. Each block consisted of standing rest for durations of about 15 s, walking the distance back and forth at a self-selected pace for about 30 s, and another standing rest period for about 15 s, while NIRS recorded hemodynamics data. The average walking distance and walking speed (averaged for Block 1 and Block 2) are presented in Table 1. The walking paradigm was identical to the experiment described in [11].

### 2.7. Longitudinal Follow-Up of Physical Activity

At baseline, the participants completed a blood draw, the neuropsychology assessment and a NIRS session in which a portable NIRS system recorded cerebral hemodynamics. All participants had weekly exercise training for a duration of 12 months (Table 1, duration of sessions). This physical exercise was part of the participants’ preventive routines offered at the ÉPIC Centre by a licensed kinesiologist. Every week the participants reported their frequency, intensity and duration of physical activity in a questionnaire. Participants had two visits, one at T0 and another follow-up visit after 12 months. At the 12-month visit, the blood draw, neuropsychology assessments and NIRS recording sessions were identical to the procedure followed at T0.

### 2.8. Pulsatility Parameters

In the present study, the primary pulsatility parameter of interest is cerebral pulse amplitude, as presented by [11], and [24]. Cerebral pulse amplitude is extracted from the pulsatile component of NIRS data (Figure 1B). Cerebral pulse amplitude quantifies instantaneous expansion of the arterial walls as a result of the passing pulse pressure wave, which leads to increased oxyhemoglobin concentration [11,23,24]. As the arterial blood is saturated with oxyhemoglobin, we solely used the NIRS channels with 850 nm wavelength, which have higher sensitivity to arterial pulsatility [11,21,22,41]. Also, we explored the association between global cerebral pulse amplitude and pulse pressure. This association indicates whether cerebral pulse amplitude is an index of arterial stiffness. Pulse pressure was calculated as the difference between systolic maximum and diastolic minimum of blood pressure measured on the arm [11,22,24].

### 2.9. Analysis of the NIRS Data for Extracting Pulsatility Parameters

We used NIRS data for the source-detector separation of 2.5–5.6 cm. This source and detector separation was used due to the result in [23], who discussed that, given the proper source-detector distance, the contamination of extracerebral signals in the pulsatile component of the NIRS data is minimal, and the measurement represents brain hemodynamics. In this study, we focused on the pre- and post-walking standing rest periods wherein data were not contaminated by movement artefacts from walking [11]. The distinction between standing rest and walking periods was performed using accelerometer data. Movement artefacts were also identified and removed from the data using the HOMER2 toolbox [42], accelerometer data and a channel-by-channel visual inspection. Data analysis was performed using MATLAB 2017a (MathWorks, Natick, MA, USA). After removing saturated channels, channels of the raw NIRS data were normalized to their means [11,22,23]. Intensity data were filtered with a bandpass filter with cutoff frequencies at 0.5–5 Hz [11,22,23,24]. This frequency band is suggested by literature and has been shown to preserve cardiac-induced pulsatility and its harmonics while eliminating unwanted high- and low-frequency noises [11,22,23]. The result of this analysis is the pulsatile component of NIRS data appearing as heartbeat epochs, which are used to extract cerebral pulse amplitude [11,22,23,24]. As a quality check for these heartbeat epochs, we used a method suggested by [43]. They showed that a good source-detector coupling will present a prominent synchronized cardiac oscillation in both wavelengths. Therefore, after preserving the cardiac component in both wavelengths, a cross-correlation at time lag zero shows how well two wavelengths are coupled. The resulting number is called the scalp coupling index (SCI) and serves as a quality check for each channel [11,22,43]. In the present study, based on sensitivity analyses, we solely used the channels with a high SCI (0.8 or higher), which indicates high-quality data, for further analysis [43]. We used the “FindPeak” function in MATLAB 2018 (MathWorks, Natick, MA, USA) and semiautomatically determined the local maxima (peaks) and local minima (nadirs) [11]. Using local maxima and local minima, each heartbeat epoch was separated, and the pulsatility index was calculated for each heartbeat epoch [11,22]. We estimated heart rate from the number of detected heartbeats in the NIRS data in the given time [11]. For noise reduction, pulsatility indices of each channel were divided into four quantiles, and the second and third quantiles (median quantiles, which are more robust to noise) were considered for further analysis [22]. Next, in each channel, the pulsatility indices of median quantiles were averaged to represent the pulsatility index for that channel (for channel-wise analysis). Also, in each participant the average pulsatility index across all the channels covering the prefrontal cortex and motor areas was calculated and was named the global cerebral pulse amplitude [11].

### 2.10. Data Exploration and Statistical Analysis

In order to control for each participant’s level of physical activity, we used regression to control cerebral pulse amplitude at T0 for the PASE score at T0. Likewise, cerebral pulse amplitude at T12 was controlled for PASE score at T12. Furthermore, to control for participants’ CVRF burden, we likewise controlled cerebral pulse amplitude at T0 and at T12 for the Framingham score (calculated from blood test parameters at T0 and at T12, respectively). Outliers were identified and excluded from the analyses using a generalized extreme Studentized deviate algorithm (or ‘gesd’) implemented in MATLAB 2018 [22]. In order to assess the reliability of the cerebral pulse amplitude, the correlation between block 1 and block 2 of the task was determined.

To address to our first hypothesis, we used a paired *t*-test to compare before-walking global cerebral pulse amplitude at T0 with before-walking global cerebral pulse amplitude at T12 (see Section 3.1). To address our second hypothesis, we used partial correlation to determine the association between changes in before walking global cerebral pulse amplitude with changes in cognitive score (see Section 3.3). To address our third hypothesis, we used a two-sample paired *t*-test to compare changes in cerebral pulse amplitude (before-walking versus after-walking) at T0. The same analyses were run with T12 values (Section 3.2).

We also conducted channel-wise analyses of the cerebral pulsatility index to explore the spatial distribution of cerebral pulsatility. A two-sample paired *t*-test was conducted on the pulsatility index, testing the null hypothesis that there was no statistically significant difference in the measured cerebral pulse amplitude for this channel. The *t*-values of each comparison were then corrected for multiple comparison with a false discovery rate (FDR) approach [44]. Along with the pulsatility index, the *t*-values that survived the FDR correction were then projected on the cortical layer of the Colin27 template using Atlas Viewer [45] in the HOMER2 [42] toolbox and some in-house scripts.

## 3. Results

On average, a block consisted of a standing rest before walking (BW) for the following durations BW_T0 (15.20 ± 6.39 s) and BW_T12 (15.50 ± 7.21 s). The standing rest periods were followed by free low-intensity walking T0 (29 ± 9.2 s) and T12 (32 ± 2.18 s). Finally, another standing rest was performed after walking (AW) for durations AW_T0 (15.77 ± 7.3 s) and AW_T12 (16.01 ± 5.12 s). The correlation between the cerebral pulse amplitude for block 1 and block 2 was calculated as follows: BW_T0 (*r* = 0.86, *p* < 0.001) and BW_T12 (*r* = 0.81, *p* < 0.001). For after-walking, this correlation between blocks was AW_T12 (*r* = 0.79, *p* < 0.01) and AW_T12 (*r* = 0.77, *p* = 0.009). These results indicated that cerebral pulse amplitude is a relatively reliable measure. Hence, for the rest of the analyses, cerebral pulse amplitude was averaged for blocks 1 and 2.

Our data indicated that pulse pressure (PP) reduced after 12 months of physical activity from T0_PP (54.89 ± 11.37) to T12_PP (49.47 ± 7.73), and this reduction was statistically significant (*p* < 0.001). Cerebral pulse amplitude was statistically significantly associated with pulse pressure for standing rest before walking, BW_T0 (*r* = 0.42, *p* = 0.006) and BW_T12 (*r* = 45.1, *p* = 0.006) and for standing rest after short-duration walking: AW_T0 (*r* = 0.47, *p* < 0.001) and AW_T12: (*r* = 49.1, *p* = 0.007).

### 3.1. Impact of 12 Months of Physical Activity on Cerebral Pulse Amplitude

**Figure 2:**
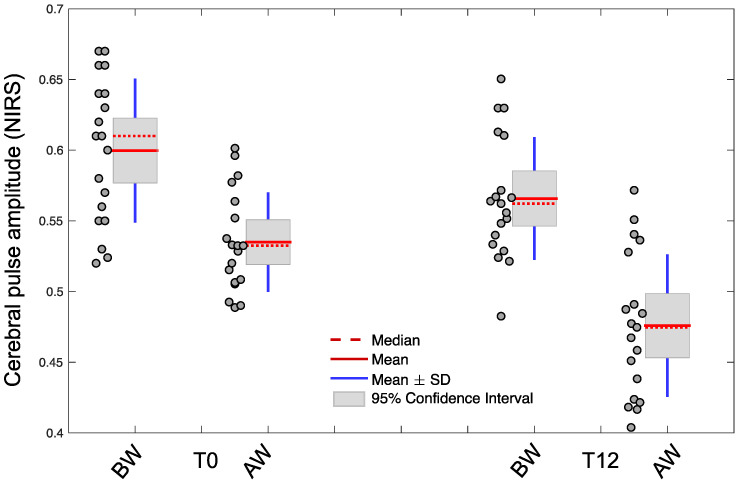
The boxplots of global cerebral pulse amplitude (averaged across all the channels) for before walking at baseline (T0) and after 12 months (T12). BW, AW and SD are abbreviations for standing rest before walking, standing rest after walking and standard deviation respectively.

Figure 2 shows the boxplots of global cerebral pulse amplitude (averaged across all the channels for each participant) for standing rest before walking and standing rest after walking in older adults with CVRF. Descriptive statistics indicated that before walking global cerebral pulse amplitude reduced with 12 months of physical activity from BW_T0_cerebral pulse amplitude (0.61 ± 0.07) to BW_T12_cerebral pulse amplitude (0.55 ± 0.06). A two-sample paired *t*-test indicated that this reduction is statistically significant (*p <* 0.001).

**Figure 3:**
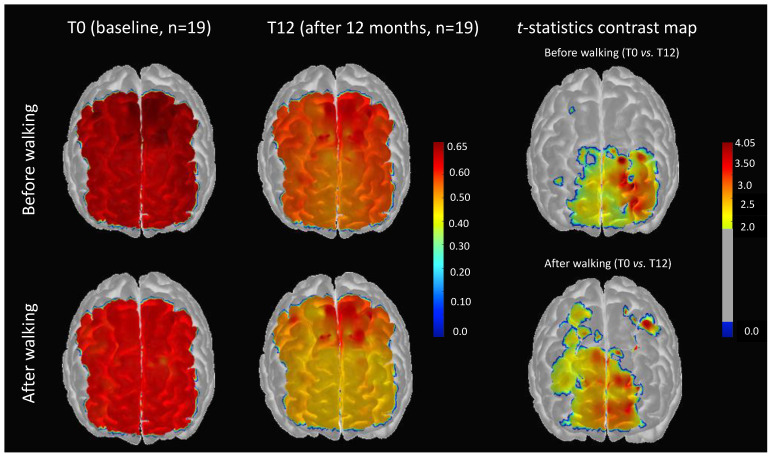
(**Left**) Channel-wise spatial distribution of the cerebral pulse amplitude projected on the cortex Colin27 template at baseline (T0) and after 12 months of physical activity (T12). (**Right**) *t*-statistics topographic contrast maps for the channel-wise comparison of cerebral pulse amplitude. These maps are thresholded and are FDR-corrected (*p* < 0.05).

Figure 3 (after walking/t-statistics contrast map) shows a channel-wise comparison of cerebral pulse amplitude for standing rest before walking for T0 versus T12. These maps are FDR-corrected (*p <* 0.05). In this figure, we can see that the reduction in cerebral pulse amplitude after 12 months of physical activity was localized in the subregions of the left prefrontal cortex (contralateral to the dominant hand) and the right superior frontal cortex.

### 3.2. Impact of 12 Months of Physical Activity on Changes of Cerebral Pulse Amplitude Immediately after Short-Duration Walking

Descriptive statistics indicated that, after short-duration walking, cerebral pulse amplitude reduced, compared with before-walking: BW_T0_cerebral pulse amplitude (0.61 ± 0.07) and AW_T0_cerebral pulse amplitude (0.57 ± 0.27). A paired *t*-test indicated that this reduction is statistically significant (*p* < 0.001). The same was determined for T12 as follows: BW_T12_cerebral pulse amplitude (0.55 ± 0.12) and AW_T12_cerebral pulse amplitude (0.47 ± 0.09), and this effect was statistically significant (*p <* 0.001). Importantly, descriptive statistics show that the magnitude of the reduction for cerebral pulse amplitude (before-walking minus after-walking) was larger at T12, and this effect was statistically significant (*p* < 0.001).

Figure 3 (after-walking/t-statistics contrast map T0 vs. T12) after 12 months of physical activity, the effect of short duration walking on cerebral pulse amplitude was localized in the right and left prefrontal cortex subregions, which, compared to the before-walking contrast map, was extended to the right precentral and right supplementary areas.

### 3.3. Relationship Between Reduction in Cerebral Pulse Amplitude and Behavioral Cognitive Performance after 12 Months of Physical Activity

Table 2 (Section 2.3) summarizes the results of four conditions of the Stroop test at time points T0 and T12. As the table indicates, 12 months of physical activity was associated with, on average, a 4.16 s reduction in the response time for the inhibition condition (condition 3), and this reduction is statistically significant (*p* = 0.008). In addition, 12 months of physical activity was associated with on average 6.42 s reduction in the response time for the switching condition, and this effect was statistically significant *(p* = 0.025). For other conditions of the Stroop test (naming and reading), a reduction in response time of about 1 s was seen, but this effect was not statistically significant (*p* > 0.1). Furthermore, total error at T0 versus T12 did not significantly differ (*p* > 0.1) on either condition of the Stroop test, which suggests that the improved response time was not due to a speed–accuracy trade-off.

We sought to explore whether the reductions in response time seen for the inhibition and switching conditions were associated with the reduction in pulsatility index. To do so, we subtracted the response time (T0 from T12) and likewise subtracted the pulsatility index for before-walking (T0 from T12) and then calculated the correlation between these two differences. Our data (without one outlier) indicated that the reduction in pulsatility index with 12 months of physical activity was statistically significantly associated with the reduction in response time for inhibition (*r* = 0.47, *p* = 0.0048) and switching (*r* = 0.49, *p* = 0.037) conditions of the Stroop test.

## 4. Discussion

This study explored the impact of physical activity on an optical index of cerebral pulsatility extracted from the pulsatile component of NIRS data in older adults with CVRF. The study has three main results: (1) Physical activity for a duration of 1 year is associated with a reduction in cerebral pulsatility index. (2) Reduction in cerebral pulsatility index is associated with improvement in cognitive performance in the executive domain. (3) Cerebral pulsatility index reduced after short-duration walking, and this reduction was greater in a 1-year follow-up.

### 4.1. Cerebral Pulse Amplitude Is a Proxy Index of Arterial Stiffness

Our data indicated that global cerebral pulse amplitude was positively associated with pulse pressure (measured from the difference between systole and diastole blood pressure) in CVRF individuals. This result corresponds to findings from [24], who reported that cerebral pulse amplitude rises with aging, and this increase is correlated with increased pulse pressure. Pulse pressure is a surrogate marker of arterial stiffness and is moderately correlated to PWV, the gold standard measure of arterial stiffness [46,47].

NIRS pulsatility data relies on changes in oxyhemoglobin concentration, which depend on the changes of the arterial pressure wave during the cardiac cycle [24]. Moreover, NIRS also relies on the ability of the arterial wall to expand and accommodate the volume of oxygenated blood [11,22]. Hence, cerebral pulse amplitude is influenced by the stiffness of the arteries in the region probed by the NIRS source and detector [11,21,22]. This is in agreement with the simplified Windkessel model presented in [11,48].

### 4.2. Impact of 12 Months of Physical Activity on Cerebral Pulsatility Index

Our data indicated a reduction in the global cerebral pulse amplitude when before-walking cerebral pulse amplitude was compared between T0 and T12. This result indicates that 12 months of regular physical activity is associated with reduced cerebral pulsatility index (lower cerebral pulse amplitude) and reduced pulse pressure. Hypothetically, the regular physical activity reduced arterial stiffness in the central arteries (for review: [49]) and improved functions of the arterial walls in these arteries [50,51] by which central pulse pressure is reduced [52] and more pulsatility is damped while the blood travels to cerebral microcirculation (which are NIRS measurement sites). Also, it is likely that regular physical activity reduced vascular stiffness, not only in the central arteries, but also in the cerebral microcirculation (such as the cerebral arterioles). A reduction of vascular stiffness in the region probed by the NIRS source and detector can improve the ability of cerebral arterial walls to expand and accommodate a higher volume of oxygenated blood in each cardiac cycle [22]. This higher volume of oxygenated blood changes the NIRS signal intensity and the pulsatility index. In summary, we suggest that, hypothetically, the combination of a reduction in the stiffness of the arterial tree in the central arteries and in the cerebral microcirculation, together with higher damping of pulsatility, could manifest lower cerebral pulse amplitude. Notably, physical exercise can influence neurohormonal mechanisms and reduce inflammation in the body [53], the mechanisms by which the arterial stiffness [54] and therefore, pulsatility may change [11]. However, such exploration is beyond the scope of the present study.

### 4.3. Impact of 12 Months of Physical Activity on Changes in the Cerebral Pulsatility Index Immediately after Short-Duration Walking

Our data indicated that short-duration walking reduced the cerebral pulse amplitude at T0 and T12. Furthermore, this reduction was statistically significantly higher at T12 than at T0. The first part of the results, which indicate at baseline (T0) that the cerebral pulse amplitude declines immediately after short-duration walking, likely shows the temporary impact of physical activity on the pulsatility index. It is possible, that due to the vasodilation of the cerebral arteries following short-duration walking, the pulsatility index is reduced [11]. However, the second part of the results shows that this reduction in cerebral pulse amplitude, which is greater after 12 months of physical activity, is likely due to the effect of long-term adaptations of vasculature to regular physical activity. It is possible that the regular physical activity for 12 months improved the responses of the vasculature to pulsatility. One such response could be the improvement in arteries’ vasodilation capacity. This interpretation is in agreement with other studies that reported that regular physical exercise enhances arterial endothelium-dependent vasodilation both in normotensive adults and those with CVRF [55]. For instance, Goto and colleagues reported that moderate to intensive physical training induces relaxation in the arterial walls through an increase in nitric oxide (NO) bioavailability [56].

Regarding a channel-wise spatial comparison of cerebral pulse amplitude, our data indicated that the reduction in cerebral pulse amplitude after 12 months of physical activity was localized in the right and left anterior prefrontal areas. These regions are territories of the middle cerebral arteries and are reported in the literature to exhibit a reduced pulsatility index with regular physical exercise [57]. Notably, after walking, this effect was extended to the right precentral and right supplementary areas. These results indicate that short-duration walking expanded the regions of channel-wise contrast.

### 4.4. Reducing Cerebral Pulsatility Index Is Associated with Improving Executive Functions

Our data indicated that reducing cerebral pulsatility index, along with 12 months of regular physical activity, is associated with reducing response time for executive functions as assessed by the Stroop test. This result agrees with other studies that reported physical-exercise-induced improvements in executive-function performance [58,59,60,61]. The improved inhibition and task switching through physical activity is of interest as these cognitive domains are reported to be impaired in aging populations [62]. In the literature, chronically higher flow pulsatility is associated with cerebral microvascular damage and impairment in the metabolism of the neurons [5,22,63,64]. Hence, it is possible that regular physical activity improved the functions of the arterial walls, by which more pulsatility is cushioned as the blood travels towards to cerebral circulation, which reduces the pulsatility that reaches the neurons, and this may support cognitive functions. Nevertheless, the present study was not designed to establish a direct cause-and-effect link between the reduction in pulsatility and improving cognitive functions.

### 4.5. Limitations

There are several limitations to the present study (1) We discussed that reducing cerebral pulsatility index in CVRF adults after 12 months of physical activity is likely due to reduced arterial stiffness in the regions we probed by NIRS. Hence, a local index of cerebral arterial stiffness, such as middle cerebral artery with PC-MRI or transcranial Doppler can support our finding. (2) Our experimental setup did not include the measurement of end-tidal CO_2_. This measure can help to explore whether the reduction in the cerebral pulsatility index after short-duration walking is associated with increases in vasodilatory substances such as CO_2_. (3) Regarding our study population, the number of participants (*n* = 19) is relatively small, which limits the statistical power of the study. Also, our participants were unbalanced in terms of distribution between women and men and the types of medication that the participants were using.

## 5. Conclusions

The present study had three main conclusions. (1) Physical activity for a duration of 12 months is association with a reduction in cerebral pulse amplitude. (2) A reduction in cerebral pulse amplitude is associated with improvements in executive function performance measured with the Stroop test. (3) The reduction of cerebral pulse amplitude (after short-duration walking compared with before walking) is greater after 12 months of physical activity. Further research is needed to determine whether cerebral pulse amplitude extracted from NIRS data can be used as a quantitative gauge of the effectiveness of physical exercise on cerebrovascular health and cognition.

## Figures and Tables

**Table 1:** Summary of demographic characteristics of the participants.

Characteristics	CVRF_T0 (*n* = 19)	CVRF_T12 (*n* = 19)
Female/male (*n*)	13/6	13/6
Age (years)	68.05 (4.99)	69.05 (4.99)
Resting SBP (mmHg)	124.57 (13.55)	120.5 (12.09)
Resting DBP (mmHg)	75.9 (5.68)	75.88 (6.97)
Pulse pressure (mmHg)	54.35 (11.33)	50.61 (8.93)
BMI (kg/m^2^)	26.99 (3.93)	26.81 (3.59)
Smoking, *n* (%)	4 (21.05)	4 (21.05)
Walking distance (m)	33.04 (3.90)	33.23 (5.92)
Walking speed (m·s^−1^)	1.10 (0.13)	1.10 (0.19)
***Physical activity***		
PASE score	127.20 (68.51)	124.90 (44.38)
Frequency of sessions per week	1.94 (0.76)	2.01 (0.87)
Duration of sessions (hour/week)	3.45 (2.33)	2.57 (1.52)
Intensity of physical activity *	4.31 (2.11)	4.07 (1.65)
***Blood sample parameters***		
Total-cholesterol (mmol·L^−1^)	4.47 (1.14)	4.91 (1.29)
LDL-cholesterol (mmol·L^−1^)	2.92 (1.92)	2.81 (0.65)
HDL-cholesterol (mmol·L^−1^)	1.35 (0.54)	1.64 (0.29)
***Medication therapy***		
Aspirin, *n* (%)	1 (0.05)	1 (0.05)
Beta-blockers, *n* (%)	2 (10.53)	1 (0.05)
Statin, *n* (%)	4 (21.05)	4 (21.05)
ACE-inhibitor, *n* (%)	2 (11.54)	3 (15.79)
ARA, *n* (%)	3 (15.79)	3 (15.79)

Abbreviations are mean (standard deviation); *n*: number of participants; % percent of participants; SBP: systolic blood pressure; DBP: diastolic blood pressure; BMI: body mass index; PASE: physical activity scale for older adults; (*) intensity of physical activity is in Borg scale; LDL: low-density lipoprotein; HDL: high-density lipoprotein; ACE: angiotensin converting enzyme; ARA: angiotensin antagonist receptor.

**Table 2:** Response time for four conditions of the Stroop test for the study population (*n* = 19).

Stroop Condition	Response Time (T0)	Response Time (T12)	*t*-Test **
Naming	31.52 (7.41)	29.105 (9.32)	*p* > 0.1
Reading	22.05 (3.68)	21.26 (6.41)	*p* > 0.1
Inhibition	58.21 (20.57)	54.05 (18.06)	*p* = 0.008 *
Switching	63.05 (5.68)	56.63 (18.35)	*p* = 0.025 *

Numbers shows four conditions of the Stroop test. Response times are reported in sec. The results reported as mean (standard deviation). The *t*-test shows the *p*-values for comparison of the response time between T0 and T12. The star sign or (**) shows the result of paired *t*-test results for comparison of the response time T0 versus T12, and (*) shows statistically significant difference between the values for *p* < 0.05.

## Data Availability

A limited version of the data and code is available upon request to corresponding author.
